# Prevalence and factors associated with anemia among children under five years of age in Rombo district, Kilimanjaro region, Northern Tanzania

**DOI:** 10.12688/f1000research.24707.3

**Published:** 2023-02-02

**Authors:** Innocent B. Mboya, Redempta Mamseri, Beatrice J. Leyaro, Johnston George, Sia E. Msuya, Melina Mgongo

**Affiliations:** 1 Department of Epidemiology and Biostatistics, Institute of Public Health, Kilimanjaro Christian Medical University College, Moshi, 255, Tanzania; 2 School of Mathematics, Statistics & Computer Science, University of Kwazulu-Natal, Pietermaritzburg, Kwazulu-Natal, Private Bag X01, Scottsville 3209, South Africa; 3 Community Health Department, Institute of Public Health, Kilimanjaro Christian Medical University College, Moshi, 255, Tanzania

**Keywords:** Anemia, prevalence, risk factors, under five children, Tanzania

## Abstract

**Background:**  Anemia is a severe public health problem affecting more than half of children under five years of age in low-, middle- and high-income countries. The study aimed to determine the prevalence and factors associated with anemia among children under five years of age in northern Tanzania.

**Methods:** This community-based cross-sectional study was conducted in Rombo district, Kilimanjaro region, northern Tanzania, in April 2016. Multistage sampling technique was used to select a total of 602 consenting mothers and their children aged 6-59 months and interviewed using a questionnaire. Data were analyzed using Stata version 15.1. We used generalized linear models (binomial family and logit link function) with a robust variance estimator to determine factors associated with anemia.

**Results:** Prevalence of anemia was 37.9%, and it was significantly higher among children aged 6-23 months (48.3%) compared to those aged 24-59 months (28.5%). There were no significant differences in anemia prevalence by sex of the child. Adjusted for other factors, children aged 6-23 months had over two times higher odds of being anemic (OR=2.47, 95% CI 1.73, 3.53, p<0.001) compared to those aged 24-59 months. No significant association was found between maternal and nutritional characteristics with anemia among children in this study.

**Conclusion:** Prevalence of anemia was lower than the national and regional estimates, and it still constitutes a significant public health problem, especially among children aged 6-23 months. The study recommends iron supplementation, food fortification, dietary diversification, and management of childhood illnesses interventions for mothers and children under two years.

## Introduction

In children under five years, anemia is a significant public health problem in the low-, middle- and high-income countries. The world health organization (WHO) defines anemia as a low blood hemoglobin concentration of less than 11g/dl in children under five years of age ^1, 2^. Anemia in children is a major cause of adverse health consequences such as stunted growth, impaired cognitive development, compromised immunity, disability and increased risk of morbidity and mortality ^2– 11^. Globally, about 43% of children under-five are anemic, and there is a marked variation in the prevalence of anemia between low- and middle-income countries (LMIC). Over 50% of anemic children live in LMIC ^12^, and the highest prevalence rate (78%) was reported in Ghana and the lowest (26%) in Cuba ^13, 14^. According to WHO, the African region has the highest proportion (62%) of anemic children ^12^.

A variety of factors causes anemia, but the most common cause is iron deficiency ^1, 3, 12^. Iron deficiency can result from inadequate dietary intake or poor absorption, increased needs for iron during the high growth periods, and increased iron loses due to helminths infection ^3^. Other causes of anemia can be infections like malaria, genetic makeup, and nutritional deficiencies of vitamins B12, A, C and folate ^3^. Factors associated with anemia also vary from region to region. The factors include the area of residence (whereby children living in rural areas beingmore at risk), low education level of the mother, child’s sex (high among males), child’s age (below 24 months) and history of infections, high birth order and maternal history of anemia ^1, 4, 13– 20^. Unemployment, low family income, low wealth quartile and high poverty index have also been associated with anemia in children under five ^5, 9, 15, 17^. In addition, poor breastfeeding practices and complementary feeding leads to anemia ^7, 14– 16^.

To combat anemia in children, WHO recommends combined strategies such as iron supplementation, especially to vulnerable populations, food-based approaches to increase iron intake through food fortification and dietary diversification and management of infectious diseases, particularly malaria and helminth infections ^21^. These strategies are recommended to be built into the primary health care system and existing programs such as maternal and child health, integrated management of childhood illness, adolescent health, safe motherhood, roll-back malaria, deworming and tuberculosis ^21^. Improved quality of anemia care is also among key strategies to accelerate progress towards addressing this problem ^22^. Although Tanzania is implementing these strategies ^23^, the Demographic and Health Survey (DHS) report shows no improvement in reducing anemia prevalence. For the two consecutive DHS rounds, 2010 and 2015, the prevalence of anemia was 58%. The results of the DHS show that the country is still far from reaching the set target of reducing anemia prevalence to 20% by 2020. In the Kilimanjaro region, Same District, anemia prevalence was 70% ^19^. Since studies show variations in factors that are associated with anemia, there was a need to conduct this study in the Rombo district as an important step towards evidence-based decision-making when planning for interventions. Geographically Same is semi-arid district while Rombo is located around Mount Kilimanjaro, hence having different topographic conditions.

## Methods

### Study design and setting

This study utilized data from a community-based cross-sectional study conducted in Rombo district, Kilimanjaro region, northern Tanzania in April 2016. Rombo district is one of the seven districts of the Kilimanjaro region. The study aimed to assess the nutritional status of children under five years in the district. The district is bordered to the north and east by Kenya, to the west by Siha and Hai districts and to the south by Moshi rural district. According to the 2012 national population and housing census, Rombo district had a total population of 260,963 of which 124,528 (52.3%) were females while 29,955 were children under five years of which 14,971 (50%) were females ^24^. The district’s largest population depends on agriculture, livestock keeping, small petty business, and few people are employed in the public sector. The district has 43 health facilities: 2 hospitals, four health centers and 37 dispensaries ^25^.

### Study population, sample size, and sampling

The study included consenting mothers and their children aged 6–59 months. A single proportion formula was used for sample size calculation. Using a standard normal value of 1.96 under 95% confidence interval, a 48% prevalence of anemia among children 6–59 months in Kilimanjaro region ^2^, a margin of error of 5% and multiplying by a design effect of 1.5 to account for cluster design, the minimum required sample size was 575 mother-child pairs.

**Figure 1.**
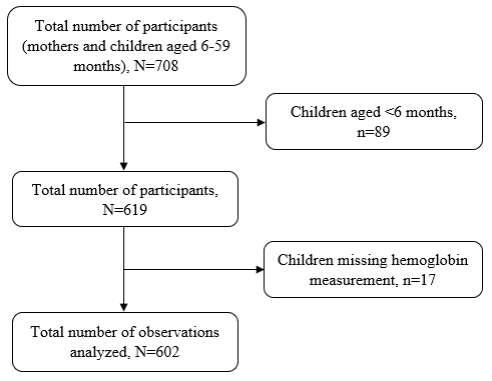
Schematic diagram showing the number of participants.

Multistage sampling technique was used to select 708 mother-child pairs from households with children aged 6–59 months. Two villages were randomly selected from each randomly selected ward. A listing of households with children under five years was generated with the help of village leaders or link persons, followed by a random selection of households. Systematic random sampling was used to select households. When the visited household had no child under five years of age, the next household was selected until the minimum required sample size was reached. If there were more than one child aged 6–59 months, the younger one was selected to represent the rest of the children in the household. If the child’s mother was not at home, the research team visited the house a minimum of three times before declaring that the participant could not be reached. Children whose mothers were not available on the day of data collection were excluded from the study as it was not possible to verify child information if next in kin or neighbor was interviewed. In addition, after excluding 89 children aged <6 months and 17 with missing hemoglobin concentrations, we analyzed data for 602 mothers-child pairs Figure 1.

### Data collection methods

A questionnaire, shared as extended data ^[Bibr ref-26]^, was used to collect data during face-to-face interviews. Although the questionnaire has not been validated in Tanzania, we adopted questions from the DHS and added some from previous literature. The following information was collected; maternal reproductive health, breastfeeding history, feeding patterns, initiation of complementary feeding, use of health facilities during pregnancy and child nutrition status. The questionnaire was in both English and Swahili languages but administered using the Swahili language, a language spoken by all the local people in this setting. Trained medical student at the Kilimanjaro Christian Medical University College collected data under the Institute of Public Health supervision.

### Study variables and measurements 

The dependent variable in this study was anemia. Anemia was defined as a blood hemoglobin concentration below 11.0 g/dl in children under five years of age ^1^. Blood samples were drawn among children from a drop of blood taken from a finger prick or heel prick (for children aged 6–11 months) and collected in a microcuvette strip. Hemoglobin (Hb) was measured on-site using a portable HemoCue rapid testing method (HemoCue ^®^ Hb 301 Analyzer - HemoCue AB, Kuvettgatan 1, SE-262 71 Angelholm, Sweden). The anemia results were given on-site and children with severe anemia (hemoglobin level <7 g/dL) were referred to the nearby health facilities.

The independent variables included socio-demographical characteristics such as age of the mother in years (<20, 20–29 and 30+), education level, occupation (Peasant/farmer, Employed and Others), marital status (single, married/cohabiting and divorced/ separated/ widowed), area of residence (rural and urban depending on how the locals define them), alcohol consumption (Yes and No), body mass index (BMI) of the mother (underweight (<18.5Kg/m ^2^); normal weight (18.5–24.9 Kg/m ^2^), overweight (25–29.9 Kg/m ^2^), and obese (≥30 Kg/m ^2^)); and child’s age and sex. Nutritional characteristics included exclusive breastfeeding (Yes and No) ^27^, colostrum feeding (Yes and No), meal frequency per day (≤3 meals and >3 meals), age at initiation of complimentary feeding (<6 months and 6+ months), and use of deworming drugs past six months (Yes and No). 

Measurement of weight was performed using a SECA weighing scale (SECA GmbH & Co. KG, Hamburg, Germany) while recumbent length was measured for children aged <24 months and standing height was measured for older children using stadiometers. At least two measurements were taken then the average was calculated. Stunting, wasting, and underweight (height-for-age, weight-for-height z-score, and weight-for-age z-score below minus two standard deviations (-2 SD), respectively) from the median of the WHO reference population ^2^. Child anthropometric z-scores were calculated using the 2006 WHO child growth standards through the “zscore06” package in Stata ^28^.

### Ethical consideration

Ethical approval was obtained from Kilimanjaro Christian Medical University College Research and Ethics Review Committee (KCMU-CRERC). Permission to conduct the study was also sought from the Rombo District Authority. Before data collection, logistics meetings were held with ward and village leaders of selected sites to inform them about the study’s purpose. The study purpose was explained to mothers before enrolment. Those who agreed to participate provided written informed consent. Unique identification numbers were used to ensure the anonymity of participant information.

### Statistical analysis

Data were analyzed using Stata version 15.1, StataCorp LLC. Means and standard deviations were used to summarize numeric variables while frequency and percentages for categorical variables. Chi-square (χ ^2^) test was used to compare the prevalence of anemia by participant characteristics. Odds ratio (OR) and 95% confidence intervals (CIs) were used to determine factors associated with anemia in children using generalized linear models (GLM) with binomial family and logit link function adjusted for potential confounding. Akaike information criteria (AIC) was used to select the best model. The GLM model with binomial family and log link function was favored against the log-linear model, i.e., Poisson family with log link function hence all the analyses were performed using the former model. A robust variance estimator was used to account for model misspecification hence improving precision of estimates. The stepwise regression method was used to select variables included in the adjusted analysis at the 10% threshold level. The age of the child remained the only significant predictor of anemia at this stage. Maternal age, alcohol use (statistically significant in the crude analysis), sex of the child, and child’s nutritional characteristics, specifically exclusive breastfeeding, wasting, stunting, and underweight, were considered potential confounders, hence included in the final model.

## Results

### Background characteristics of mothers and children

Data were analyzed for a total of 602 mothers and children aged 6–59 months. The mean age (SD) of mothers in this study was 29.9±7.6 years. More than half (52%) of all mothers were aged between 20–29 years, 70% had primary school education level, 81.3% were married or cohabiting with their partners. The prevalence of obesity among women was 14.3%. The median age (IQR) of children in this study was 24 (14, 36) months while more than half (52.5%) were aged between 24–59 months. Also, more than half (52.7%) of all children were males Table 1 ^29^.

**Table 1.** Background characteristics of mothers and children (N=602).

Variables	Frequency	Percentage
**Age categories of the mother in** years *		
Mean (SD)	29.9 (7.6)	
<20	19	3.2
20–29	307	52.0
30+	264	44.8
Education level *		
None	13	2.2
Primary	420	69.9
Secondary and above	168	28.0
Marital status *		
Single	73	12.2
Married/Cohabiting	487	81.3
Divorced/ separated/ widowed	39	6.5
Occupation *		
Peasant/farmer	366	64.9
Employed	160	28.3
Others	38	6.7
**Area of residence**		
Urban	27	4.5
Rural	575	95.5
Body mass index categories *		
Normal	285	47.9
Underweight	26	4.4
Overweight	199	33.4
Obese	85	14.3
**Consume alcohol**		
No	367	60.9
Yes	235	39.0
**Attended ANC during pregnancy for** this baby *		
No	13	2.2
Yes	585	97.8
Number of ANC visits * (n=585)		
≥4	382	65.8
<4	199	34.2
**Sex of the child**		
Male	317	52.7
Female	285	47.3
**Age of the child (months)**		
Median (IQR)	24 (14, 36)	
6–23	286	47.5
24–59	316	52.5

*Variable with missing information.

### Feeding practices and nutritional status of children

The vast majority (96.3%) were given colostrum while the overall prevalence of exclusive breastfeeding up to six months was 40.1%. Less than half (45.2%) of children in this study were given more than three meals per day and 69.7% were initiated complimentary feeding before six months. Also, 70.5% of children in this study were given deworming drugs. This study’s prevalence of wasting, stunting, and underweight was 10%, 38.5%, and 6%, respectively Table 2 ^29^.

**Table 2.** Nutritional characteristics (N=602).

Variables	Frequency	Percentage
Child given deworming drugs *		
No	171	29.5
Yes	408	70.5
Baby given colostrum *		
No	22	3.7
Yes	577	96.3
Meal frequency per day *		
≤3	321	54.8
>3	265	45.2
Age at complementary feeding *		
<6 months	375	69.7
≥6 months	163	30.3
Child exclusively breastfed *		
No	349	59.9
Yes	234	40.1
**Wasted**		
No	542	90.0
Yes	60	10.0
**Stunted**		
No	370	61.5
Yes	232	38.5
**Underweight**		
No	566	94.0
Yes	36	6.0

*Variable with missing information

### Prevalence of anemia by child’s age and sex

**Figure 2.**
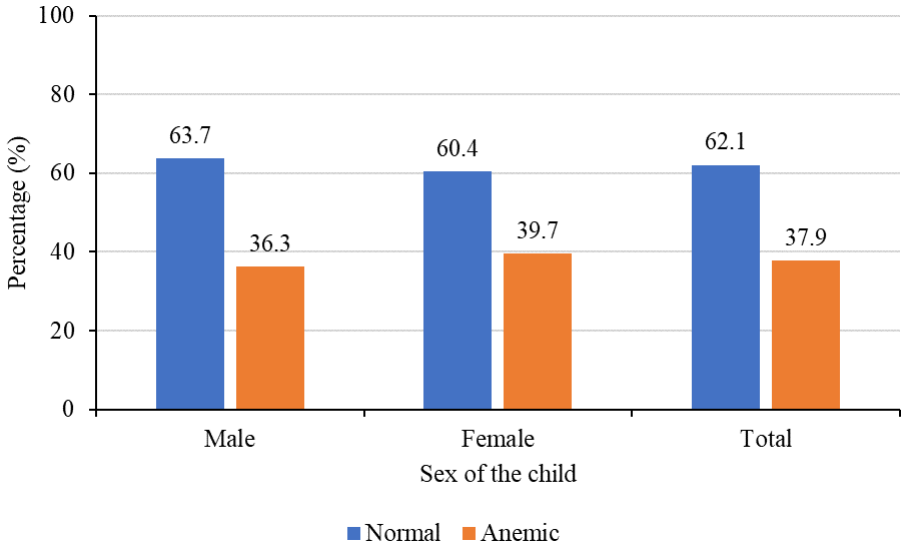
Prevalence of anemia by sex of the child (N=602).

**Figure 3.**
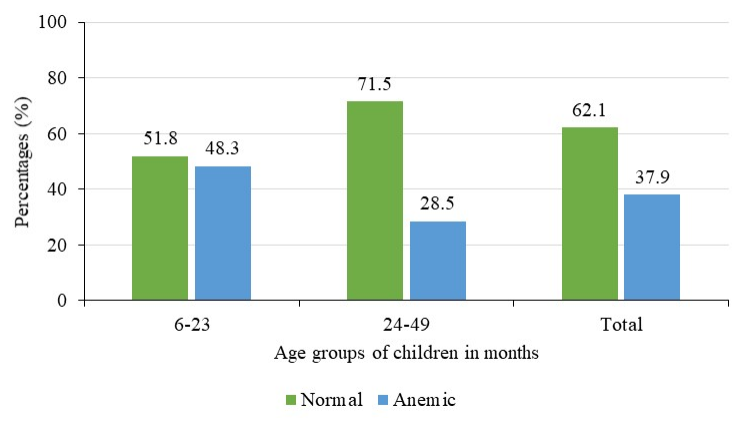
Prevalence of anemia by age groups of children in months (N=602).

In this study, the mean (SD) hemoglobin level of children aged 6–59 months was 11.2±1.6g/dl and the prevalence of anemia (hemoglobin level less than 11g/dl) was 37.9%. Prevalence was slightly higher among females (39.7%) compared to 36.2% among males Figure 2 ^29^, but this difference was not significant (p=0.40). Prevalence was much higher among children aged 6–23 months (48.1%) compared to 28.5% among those aged 24–59 months Figure 3 ^29^. These differences in the prevalence by age were statistically significant (p<0.001).

### Factors associated with anemia

The study performed crude and adjusted analyses to determine factors associated with anemia in children aged 6–59 months. In the crude analysis, factors associated with anemia were whether the mother consumed alcohol, exclusive breastfeeding, and child’s age Table 3 ^29^. Lower odds of anemia were observed among children whose mothers consumed alcohol (OR=0.68, 95%CI 0.48, 0.95, p=0.03). Higher odds of anemia were observed among children who were breastfed exclusively (OR=1.53, 95%CI 1.09, 2.14, p=0.02) and children aged 6–23 months (OR=2.34, 95%CI 1.67, 3.28) compared to those aged 24–59 months which showed a much stronger association with anemia (p<0.001). There was a positive association between stunting and the odds of anemia (OR=1.39, 95%CI 0.99, 1.95) but this association was not strong (p=0.06), Table 3 ^29^.

**Table 3.** Crude analysis for factors associated with anemia in children under five (N=602).

Variables	N	Anemic (%)	COR *	95% CI	p-value
**Age categories of the mother in** **years**					
<20	19	9 (47.4)	1.58	0.62, 4.01	0.34
20–29	307	119 (38.8)	1.12	0.79, 1.56	0.56
30+	264	96 (36.4)	1.00		
**Education level**					
None	13	3 (23.1)	0.45	0.12, 1.70	0.24
Primary	420	158 (37.6)	0.91	0.63, 1.31	0.61
Secondary+	168	67 (39.9)	1.00		
**Marital status**					
Single	73	29 (39.7)	1.09	0.66, 1.80	0.80
Married/Cohabiting	487	184 (37.8)	1.00		
Divorced/ separated/ widowed	39	14 (35.9)	0.92	0.45, 1.74	0.82
**Occupation**					
Peasant/farmer	366	127 (34.7)	0.72	0.49, 1.05	0.09
Employed	160	68 (42.5)	1.00		
Others	38	21 (55.3)	1.67	0.82, 3.41	0.16
**Body mass index categories**					
Normal	285	116 (40.7)	1.00		
Underweight	26	10 (38.5)	0.91	0.40, 2.08	0.82
Overweight	199	69 (34.7)	0.77	0.53, 1.13	0.18
Obese	85	32 (37.7)	0.88	0.53, 1.45	0.61
**Consume alcohol**					
No	367	152 (41.4)	1.00		
Yes	235	76 (32.3)	0.68	0.48, 0.95	**0.03**
**Number of ANC visits**					
≥4	382	148 (38.7)	1.00		
<4	199	73 (36.7)	0.92	0.64, 1.31	0.63
**Child given deworming drugs**					
No	171	72 (42.1)			
Yes	408	150 (36.8)	0.80	0.56, 1.15	0.23
**Baby given colostrum**					
No	22	10 (45.5)	1.00		
Yes	576	215 (37.3)	0.71	0.30, 1.68	0.44
**Meal frequency per day**					
≤3	321	118 (36.7)	1.00		
>3	265	105 (39.6)	1.13	0.81, 1.58	0.48
**Age at complementary feeding**					
<6 months	375	137 (36.5)	1.00		
≥6 months	163	71 (43.6)	1.34	0.92, 1.95	0.13
**Child exclusively breastfed**					
No	349	120 (34.4)	1.00		
Yes	234	104 (44.4)	1.53	1.09, 2.14	**0.02**
**Wasted**					
No	542	207 (38.2)	1.00		
Yes	60	21 (35.0)	0.87	0.50, 1.52	0.63
**Stunted**					
No	370	129 (34.8)	1.00		
Yes	232	99 (42.7)	1.39	0.99, 1.95	0.06
**Underweight**					
No	566	217 (38.3)	1.00		
Yes	36	11 (30.6)	0.71	0.34, 1.47	0.35
**Sex of the child**					
Male	317	115 (36.2)	1.00		
Female	285	113 (39.7)	1.15	0.83, 1.61	0.40
**Child age categories**					
6–23	286	138 (48.1)	2.34	1.67, 3.28	**<0.001**
24–59	316	90 (28.5)	1.00		

*COR=Crude odds ratio

Adjusted analysis for factors associated with anemia in children is shown in Table 4 ^29^. A multivariable model was developed by adding and later removing one variable after another to assess the presence and effect of confounding. Age of the child was the only variable that remained to be strongly (p<0.001) associated with higher odds of anemia. Adjusted for mother’s age categories (years), whether a mother consumed alcohol during pregnancy, exclusive breastfeeding, wasting, stunting and child’s sex, children aged 6–23 months had over two times higher odds of being anemic (OR=2.47, 95%CI 1.73, 3.53) compared to those aged 24–59 months Table 4 ^29^.

**Table 4.** Adjusted analysis for factors associated with anemia in children under five (N=602).

Variables	AOR *	95% CI	p-value
**Age categories of the mother in** **years**			
<20	0.71	0.27, 2.84	0.48
20–29	0.86	0.59, 1.24	0.42
30+	1.00		
**Consume alcohol**			
No	1.00		
Yes	0.70	0.48, 1.02	0.06
**Child exclusively breastfed**			
No	1.00		
Yes	1.38	0.97, 1.98	0.08
**Wasted**			
No	1.00		
Yes	0.86	0.43, 1.72	0.66
**Stunted**			
No	1.00		
Yes	1.40	0.97, 2.02	0.07
**Underweight**			
No	1.00		
Yes	0.99	0.39, 2.52	0.98
**Sex of the child**			
Male	1.00		
Female	1.01	0.71, 1.44	0.94
**Child age categories**			
6–23	2.47	1.73, 3.53	<0.001
24–59	1.00		

*AOR: Adjusted odds Ratio

## Discussion

The prevalence of anemia among children aged 6–59 months in this study was 37.9%. Age of the child was the only factor significantly associated with anemia among children. This study’s prevalence of anemia in this study is much lower than the national and regional estimates ^2^ and other sub-population studies in Tanzania ^9, 19^. One of these studies was hospital-based ^9^, while the other included children aged 1–35 months ^19^ that could explain the differences. Prevalence in this study is also lower than those reported in other countries ^5, 13, 15, 16, 30^. High prevalence in other studies could be linked to differences in the study population and wider population coverage since most utilized nationally representative data such as DHS data. A study by Ayoya *et al*. observed a similar prevalence (39%) among under-five children in Haiti ^4^. Pita *et al*. observed a lower (26%) prevalence in Cuba ^14^, which may be due to food-fortification interventions among other strategies ^14^. Despite the observed differences, the prevalence reported in this study constitutes a significant public health problem ^12^ that needs intensified efforts.

In this study, adjusted for the background and nutritional characteristics, children aged 6–23 months had higher odds of having anemia than those aged 24–59 months. Infants (<24 months) are consistently reported to be at higher odds of being anemic in other studies ^2, 4, 5, 13, 14, 31, 32^. Infants have a higher demand for nutrients needed for their growth, hence need proper complementary feeding. In this setting, there is a practice of giving porridge (a mixture of water, maize flour, and added sugar), cow’s milk and less diversified foods at a younger age ^33^. This practice could be one of the factors that leads to poor anemia status in children ^33, 34^. Also, conflicting advice on infant and young child feeding from various sources, including close relatives, community members, and health care providers affects breastfeeding practices, impacting the child’s anemia status ^34^. Receiving quality anemia care, particularly nutrition advice about healthy foods and the minimum acceptable diet to the care giver, and routine hemoglobin measurement is critical in reducing anemia burden for children 6–23 months, who are most at risk ^22^.

There were no significant differences in the prevalence of anemia by sex of the child in this study which is consistent with findings from other studies ^13, 14, 18, 30^. On the contrary, females have been reported to be less likely to be anemic in Ethiopia ^16^, which is contrary to findings from Kenya where the risk was high in male children (aged 6 months to 14 years) ^31^, which could account for these differences. We did not find an association between maternal characteristics such as age categories, education level, occupation and ANC visits among others contrary to other studies. ANC visit and mother’s occupation have been associated with anemia elsewhere ^7, 16^. The higher education level of mothers is protective against childhood anemia ^15, 19, 31^.

Likewise, there was no association between nutritional characteristics such as deworming drugs uptake, exclusive breastfeeding (EBF), colostrum feeding, complementary feeding, and feeding frequency with anemia. However, other studies reported an association between nutritional characteristics with a higher risk of anemia in under-five children ^4, 5, 14, 18, 33^. On the contrary, Meinzen-Derr *et al.* ^20^ reported that, infants exclusively breastfed for six months in developing countries might be at increased risk of anemia, especially among mothers with poor iron status. In addition, there is evidence that the longer the infant is exclusively breastfed, the worse the severity of childhood anemia due to low iron content in breast milk ^35, 36^. The positive association between EBF and anemia was observed in this study but was not statistically significant. The effect of EBF on anemia in children is an area that needs further research. Despite the observed association in this study, nutritional interventions (EBF included) are among the key strategies to reduce the burden of anemia in under-five children ^21, 23, 27^.

The study involved participants from most wards in the Rombo district, providing a picture of anemia in children under five. However, the findings in this study may not be generalized to other districts in Kilimanjaro and regions across the country. Also, the study might have been prone to recall and social desirability bias due to the self-reporting of nutritional practices associated with anemia. These may under or over-estimate these practices in the district.

## Conclusion

The prevalence of anemia was lower than the national and regional prevalence but it still constitutes a significant public health problem especially among children aged 6–23 months. There were no significant differences in anemia prevalence by sex of the child and any of the nutritional characteristics. The study recommends iron supplementation, food fortification, dietary diversification, and management of childhood illnesses interventions for mothers and children under two years. Future studies should apply mixed methods, including longitudinal follow-up, to explore and determine the factors associated with anemia in children necessary to inform context-specific interventions.

## Data Availability

Harvard Dataverse: Anaemia in children under five years of age in rural Tanzania. https://doi.org/10.7910/DVN/KJMNID ^29^ This project contains the following underlying data:
- anemiaU5_rombo2016data.tab (Data on anaemia prevalence and associated factors among children under five years of age in the Rombo district, Kilimanjaro region, Northern Tanzania) Data are available under the terms of the Creative Commons Zero "No rights reserved" data waiver (CC0 1.0 Public domain dedication). Figshare: Questionnaire: Nutritional status of children U5 years of age in Kilimanjaro Region, Northern Tanzania. https://doi.org/10.6084/m9.figshare.12553844.v2 ^26^ This project contains the following extended data:
- Questionnaire - Nutritional status of children U5 years of age - English.pdf (Study questionnaire - English) - Questionnaire - Nutritional status of children U5 years of age.pdf (Study questionnaire) Data are available under the terms of the Creative Commons Attribution 4.0 International license (CC-BY 4.0).
