# Supplementary material for: Prevalence and factors associated with anemia among children under five years of age in Rombo district, Kilimanjaro region, Northern Tanzania
Source: F1000Res. 2023 Feb 2;9:1102. Originally published 2020 Sep 7. [Version 3] doi: 10.12688/f1000research.24707.3 (PMC9936566; doi:10.12688/f1000research.24707.3)
Supplement: Peer review history

# Peer review: Reviewer response for version 3

**DOI:** 10.5256/f1000research.142659.r162190

**Published:** 2023-02-15

**Authors:** Mala Ali Mapatano

**Affiliations:** 1 Kinshasa School of Public Health, University of Kinshasa, Kinshasa, Kinshasa, Congo

I am satisfied and approve the publication.

Is the work clearly and accurately presented and does it cite the current literature?

Partly

If applicable, is the statistical analysis and its interpretation appropriate?

Yes

Are all the source data underlying the results available to ensure full reproducibility?

Yes

Is the study design appropriate and is the work technically sound?

Yes

Are the conclusions drawn adequately supported by the results?

Yes

Are sufficient details of methods and analysis provided to allow replication by others?

Yes

Reviewer Expertise:

NA

I confirm that I have read this submission and believe that I have an appropriate level of expertise to confirm that it is of an acceptable scientific standard.

# Peer review: Reviewer response for version 2

**DOI:** 10.5256/f1000research.78635.r146334

**Published:** 2022-09-07

**Authors:** Mala Ali Mapatano

**Affiliations:** 1 Kinshasa School of Public Health, University of Kinshasa, Kinshasa, Kinshasa, Congo

**Title**: the methodology and results sections specify that the study focuses on children 6-59 months old. The title should reflect the same age category.

**Results**:

- The study shows no association with *exclusive breastfeeding*, *colostrum feeding*. These two variables are not appropriate in this context, since the methodology section limits the study to children 6-59 months.

**Discussion**:

- The authors don’t discuss thoroughly the differences between their findings and the literature:

For example:

1. *Age of the child was the only factor significantly associated with anemia among children*. What could have been the confounding factors?
2. *High prevalence in other studies could be linked to differences in the study population and wider population coverage since most utilized nationally representative data such as DHS data.* What are these differences?
3. After the statement of high prevalence in other studies, one would expect that the authors would cite those studies. Instead, they mention Ayoya et al. and
Pita et al whose findings do not reflect high prevalence of anemia.

**References**:

Reference 2: is related to the Tanzania DHS, not to a WHO report. Therefore, the DHS is not the primary source of the definition of anemia, but only used it.

Reference 3: This policy brief focuses on women of reproductive age, not on children 6-59 months of age. It may not be relevant here. In fact, it refers to the general WHO definition of anemia, without even indicating the cut-off points.

Is the work clearly and accurately presented and does it cite the current literature?

Partly

If applicable, is the statistical analysis and its interpretation appropriate?

Yes

Are all the source data underlying the results available to ensure full reproducibility?

Yes

Is the study design appropriate and is the work technically sound?

Yes

Are the conclusions drawn adequately supported by the results?

Yes

Are sufficient details of methods and analysis provided to allow replication by others?

Yes

Reviewer Expertise:

Nutritionist; epidemiologist

I confirm that I have read this submission and believe that I have an appropriate level of expertise to confirm that it is of an acceptable scientific standard, however I have significant reservations, as outlined above.

# Response

**Published:** 2022-12-27

**Authors:** Innocent Mboya

**Affiliations:** Lund University, Sweden

Dear Mala Ali Mapatano, 

 We thank you for taking the time to review our manuscript. Below are clarifications and responses to your comments. 

**Title**: the methodology and results sections specify that the study focuses on children 6-59 months old. The title should reflect the same age category.

 Response: We thank the reviewer for this comment. However, we would like the title to be stated the same way because children 6-59 months still constitute the under-five-year category. Indeed, previous studies stated the same way even though their target population was children 6-59 months [1].

**Results**:

- The study shows no association with *exclusive breastfeeding*, *colostrum feeding*. These two variables are not appropriate in this context, since the methodology section limits the study to children 6-59 months.

Response: Previous studies established an association between exclusive breastfeeding with anemia in children 0-6 months. Women in this study were asked whether they breastfed the child exclusively for six months (regardless of the child’s current age), the responses being Yes/No, but no association was observed with anemia. As indicated in the last but one paragraph of our discussion, more studies are necessary to establish a cause-effect relationship between exclusive breastfeeding and the risk of anemia in children. Nevertheless, there is evidence that the longer the infant is exclusively breastfed, the worse the severity of childhood anemia due to low iron content in breast milk [2, 3]. We have also added this information in the discussion section.

**Discussion**:

- The authors don’t discuss thoroughly the differences between their findings and the literature:

For example:

*1. Age of the child was the only factor significantly associated with anemia among children*. What could have been the confounding factors?

 Response: Several analyses were performed to determine the factors associated with anemia among children 6-59 months in this study. Details are also contained in the data analysis section. Age was the only statistically significant predictor of anemia even after adjusting for background and nutritional characteristics. We have added this description in the discussion section.

*2. High prevalence in other studies could be linked to differences in the study population and wider population coverage since most utilized nationally representative data such as DHS data.* What are these differences?

 Response: By differences here we refer to the methodological variations in the included population. Cross-sectional surveys in a small geographical area are likely to give a slight difference estimate compared to the nationally representative sample such as the DHS. The first paragraph of the discussion section specifically highlights these issues.

 3. After the statement of high prevalence in other studies, one would expect that the authors would cite those studies. Instead, they mention Ayoya et al. and

Pita et al whose findings do not reflect high prevalence of anemia.

 Response: Those studies are cited just before this statement. The sentence citing Ayoya et al is slightly edited for clarity.

**References**:

Reference 2: is related to the Tanzania DHS, not to a WHO report. Therefore, the DHS is not the primary source of the definition of anemia, but only used it.

 Response: Reference 2 is the Tanzania Demographic and Health Survey report of 2015/16 and it contains the cited information. These anthropometric measurements were also assessed following a similar methodology documented in this report. In addition, reference 1 cites the WHO document defining anemia in children 6-59 months.

Reference 3: This policy brief focuses on women of reproductive age, not on children 6-59 months of age. It may not be relevant here. In fact, it refers to the general WHO definition of anemia, without even indicating the cut-off points.

 Response: We have removed this citation of the definition of anemia in children under five.

 References

1. Obasohan PE, Walters SJ, Jacques R, Khatab K. A Scoping Review of the Risk Factors Associated with Anaemia among Children Under Five Years in Sub-Saharan African Countries. Int J Environ Res Public Health. 2020 Nov 27;17(23):8829. doi: 10.3390/ijerph17238829. PMID: 33261060; PMCID: PMC7731158.
2. Buck S, Rolnick K, Nwaba AA, Eickhoff J, Mezu-Nnabue K, Esenwah E, Mezu-Ndubuisi OJ. Longer breastfeeding associated with childhood anemia in rural south-eastern Nigeria. International Journal of Pediatrics. 2019 Jun 10;2019.
3. Burke RM, Rebolledo PA, Aceituno AM, Revollo R, Iñiguez V, Klein M, Drews-Botsch C, Leon JS, Suchdev PS. Effect of infant feeding practices on iron status in a cohort study of Bolivian infants. BMC pediatrics. 2018 Dec;18(1):1-9.

# Peer review: Reviewer response for version 2

**DOI:** 10.5256/f1000research.78635.r120374

**Published:** 2022-02-17

**Authors:** Indrapal I Meshram

**Affiliations:** 1 Division of Public Health Nutrition, National Institute of Nutrition, Hyderabad, Telangana, India

- In method: Under study design, it is mentioned that he study aimed to assess the nutritional status of children under five years in the district. but in abstract, only anemia prevalence is mentioned and also title does not reflect this under study population, it is still not clear how many wards were selected and why systematic sampling was used for selection of HHs.
- Under study variable, para 3, it is mentioned reference 2 for WHO reference population, but reference 2 is not WHO reference. 
- Under statistical analysis, nothing is mentioned about how nutritional status was decided. 
- In table 1, single mother is mentioned, how single mother has child, it may be separated/divorcee of widow, this classification whether used in Africa the prevalence of underweight mentioned is 6% and wasting 10%, i still have some doubt about this percent as stunting is 38%. While considering reference in logistic regression, always we consider good practices as reference, but this is not followed in this table. Age at Complementary feeding should be below 6 month, 6-8 months and after 8 months and likely analysis should be done.

Is the work clearly and accurately presented and does it cite the current literature?

Yes

If applicable, is the statistical analysis and its interpretation appropriate?

Yes

Are all the source data underlying the results available to ensure full reproducibility?

Yes

Is the study design appropriate and is the work technically sound?

Partly

Are the conclusions drawn adequately supported by the results?

Yes

Are sufficient details of methods and analysis provided to allow replication by others?

Yes

Reviewer Expertise:

Public Health Nutrition

I confirm that I have read this submission and believe that I have an appropriate level of expertise to confirm that it is of an acceptable scientific standard, however I have significant reservations, as outlined above.

# Response

**Published:** 2022-11-25

**Authors:** Innocent Mboya

**Affiliations:** Lund University, Sweden

Dear Dr. Indrapal I Meshram, 

 We thank you for your time in reviewing this manuscript. Please find below our responses to your recent comments with detailed clarifications. 

 In method: Under study design, it is mentioned that he study aimed to assess the nutritional status of children under five years in the district. but in abstract, only anemia prevalence is mentioned and also title does not reflect this under study population, it is still not clear how many wards were selected and why systematic sampling was used for selection of HHs.

***Response: **The current study used secondary data from a community-based cross-sectional study conducted in the district whose primary purpose was to assess the nutritional status of children under five years of age. Our analysis focused primarily on anemia and its associated factors given the high burden of this problem in the region and Tanzania in general.*

 Under study variable, para 3, it is mentioned reference 2 for WHO reference population, but reference 2 is not WHO reference. 

***Response:** We acknowledge the reviewer's comment. Please note that reference 2 is the Tanzania Demographic and Health Survey report of 2015/16 and it contains the cited information. These anthropometric measurements were also assessed following a similar methodology documented in this report.*

 Under statistical analysis, nothing is mentioned about how nutritional status was decided. 

***Response: **We have this information at the end of paragraph 3 of the study variables section which reads; “Child anthropometric z-scores were calculated using the 2006 WHO child growth standards through the “zscore06” package in Stata”. This function in Stata software allows the specification of the child’s height, weight, age, and sex among other parameters for the calculation of z-scores.*

 In table 1, single mother is mentioned, how single mother has child, it may be separated/divorcee of widow, this classification whether used in Africa the prevalence of underweight mentioned is 6% and wasting 10%, i still have some doubt about this percent as stunting is 38%. While considering reference in logistic regression, always we consider good practices as reference, but this is not followed in this table. Age at Complementary feeding should be below 6 month, 6-8 months and after 8 months and likely analysis should be done.

***Responses:***

- *Regarding marital status, it is possible for a single mother to have a child even without being married. The reason is a woman may choose to have children without any marital commitment. This classification is not specific in African settings. Existing literature documents different socio-cultural, economic, and health issues experienced by single mothers. The contextual definition of single motherhood may differ depending on the area of application. Some examples are illustrated in these two sources, one which is specific to Tanzania [1, 2].*
- *Regarding the reported results on the anthropometric measures (i.e., wasting, stunting, and underweight), we would like to indicate that our results are consistent with the Tanzania Demographic and Health Survey of 2015/16 [3]. The first paragraph of the discussion section compares the observed results.*
- *Regarding Age at complementary feeding, we want to acknowledge the reviewer comment. However, the reason for having two groups (< 6 months and ≥ 6 months) as shown in Table 2 and 3 is to gain more statistical power as a function of sample size. It can be seen in Table 2 that there were 163 (30.3%) children initiated complementary feeding at ≥ 6 months. Breaking this variable further will not improve the results observed in Table 3 and may increase the probability of committing type I error [4].*

References

1. Golombok S, Zadeh S, Freeman T, Lysons J, Foley S. Single mothers by choice: Parenting and child adjustment in middle childhood. J Fam Psychol. 2021 Mar;35(2):192-202. doi: 10.1037/fam0000797. Epub 2020 Sep 17.
2. The Citizen (2021). Surviving and thriving as single mothers. Available from; https://www.thecitizen.co.tz/tanzania/magazines/woman/surviving-and-thriving-as-single-mothers-2640448. Date Accessed; 25 November 2022.
3. MoHCDGEC, MoH, NBS, OCGS, & ICF. (2016). Tanzania Demographic and Health Survey and Malaria Indicator Survey (TDHS-MIS) 2015-16. Ministry of Health, Community Development, Gender, Elderly and Children (MoHCDGEC) [Tanzania Mainland], Ministry of Health (MoH) [Zanzibar], National Bureau of Statistics (NBS), Office of the Chief Government Statistician (OCGS), and ICF. Dar es Salaam, Tanzania and Rockville, Maryland, USA.
4. Banerjee A, Chitnis UB, Jadhav SL, Bhawalkar JS, Chaudhury S. Hypothesis testing, type I and type II errors. Ind Psychiatry J. 2009 Jul;18(2):127-31. doi: 10.4103/0972-6748.62274.

# Peer review: Reviewer response for version 1

**DOI:** 10.5256/f1000research.27253.r95218

**Published:** 2021-10-08

**Authors:** Tania Supali

**Affiliations:** 1 Department of Parasitology, Universitas Indonesia, Jakarta, Indonesia

It is difficult to review the manuscript because the format of manuscript is not in one column with line numbering.

- **Introduction:**  needs to be rewritten properly
- **Methods:**
  - It should be written systematically. Example: Data collection for Hb measurement (anemia) should be separated from collecting questionnaire data.  
  - The rural and urban study areas were not described in detail. There are 27 children from urban and 575 children from rural (Table 1). What is the definition of urban and rural? 
  - Why was the number of urban children only 27 children?
- **Results:**
  - Prevalence of anemia based on age and sex – can be combined in one table. 
  - Table 1 and 3 can be combined in one table.
  - Please rewrite the results systematically  
- **Discussion:**
  - It is unclear why children below 2 years old showed high prevalence of anemia compared to 24-59 months children. Please explain in more details.
  - A  question about deworming drugs was included in the questionnaire. What was the prevalence of STH in the study area?  The impact of intestinal helminth infection should be discussed in this manuscript.   
  - Malaria is prevalent in Tanzania. Why wasn't malaria data included in the study, given that it contributes to anemia?

In general: The manuscript needs major revision.

Is the work clearly and accurately presented and does it cite the current literature?

Partly

If applicable, is the statistical analysis and its interpretation appropriate?

Yes

Are all the source data underlying the results available to ensure full reproducibility?

Yes

Is the study design appropriate and is the work technically sound?

Partly

Are the conclusions drawn adequately supported by the results?

Yes

Are sufficient details of methods and analysis provided to allow replication by others?

Yes

Reviewer Expertise:

Parasitology

I confirm that I have read this submission and believe that I have an appropriate level of expertise to confirm that it is of an acceptable scientific standard, however I have significant reservations, as outlined above.

# Response

**Published:** 2021-10-20

**Authors:** Innocent Mboya

**Affiliations:** Lund University, Sweden

Dear Supali, 

 Thank you for your time to review this manuscript. We have addressed your comments and suggestions as detailed below. 

- *Introduction: needs to be rewritten properly*
- **Response: **The introduction has been revised accordingly.
- *Methods: It should be written systematically. Example: Data collection for Hb measurement (anemia) should be separated from collecting questionnaire data.  *
- **Response: **Information about Hb and other measurements has been shifted to the study variables and measurements section.
- *The rural and urban study areas were not described in detail. There are 27 children from urban and 575 children from rural (Table 1). What is the definition of urban and rural? *
- **Response: **As also responded to the first reviewer, Rombo is considered a rural district of Kilimanjaro region. However, participants were asked whether they considered the place of their residence as urban or rural. Those who resided in urban areas were mostly from the district’s centre (mostly referred by locals as urban or town). The key services such as the district’s commissioner’s office/headquarter, district hospital, among others are in this setting. In addition, wards are largest administrative units and contains several villages.
- *Why was the number of urban children only 27 children?*
- **Response: **Please see the response above.
- *Results: Prevalence of anemia based on age and sex – can be combined in one table. *
- **Response: **The reviewer response is acknowledged. The figures are much preferred against tables for better presentation and for the capturing the reader’s interest. This is also supported by the observed findings in this study where anemia prevalence differed significantly by child’s age groups.
- *Table 1 and 3 can be combined in one table.*
- **Response: **Tables 1 and 2 focuses on describing the participant background and nutritional characteristics. Table 3 contains slightly different information. It compares the prevalence of anemia by these characteristics and provides results from the crude regression analysis. For these reasons, we would like to retain the current table structure.
- *Please rewrite the results systematically*
- **Response: **The results section has been revised accordingly.
- *Discussion: It is unclear why children below 2 years old showed high prevalence of anemia compared to 24-59 months children. Please explain in more details.*
- **Response: **The reviewer comment is acknowledged. The first and second paragraphs of the discussion section has been revised to clearly explain the observed results, which also compare with previous studies and shows the public health implications.
- *A  question about deworming drugs was included in the questionnaire. What was the prevalence of STH in the study area?  The impact of intestinal helminth infection should be discussed in this manuscript.*
- **Response: **Unfortunately, the study did not assess the prevalence of STH which may be associated with increased anemia risk, which remains an area for future studies.
- *Malaria is prevalent in Tanzania. Why wasn't malaria data included in the study, given that it contributes to anemia?*
- **Response: **We thank the reviewer for this comment. Although malaria is prevalent in Tanzania, not all regions are highly affected. At the regional level, malaria prevalence is high in the Southern slopes of Mount Kilimanjaro compared to the highlands[1]. Secondly, the primary study aim was to assess prevalence of anemia in children under five in the Rombo district. A more comprehensive study that capture the social-economic, demographic, behavioral, and clinical determinants of anemia and other child nutritional-related characteristics is essential in this setting.
- *In general: The manuscript needs major revision.*
- **Response: **The manuscript has been revised accordingly.

[1] Kassam, N. A., Kaaya, R. D., Damian, D. J., Schmiegelow, C., Kavishe, R. A., Alifrangis, M., & Wang, C. W. (2021). Ten years of monitoring malaria trend and factors associated with malaria test positivity rates in Lower Moshi. *Malaria Journal*, *20*(1), 1-9.

# Peer review: Reviewer response for version 1

**DOI:** 10.5256/f1000research.27253.r84355

**Published:** 2021-05-17

**Authors:** Indrapal I Meshram

**Affiliations:** 1 Division of Public Health Nutrition, National Institute of Nutrition, Hyderabad, Telangana, India

**Introduction**

1. Globally, about 43% of children under-five are anemic, and **there is a marked variation in the prevalence of anemia between lowand middle-income countries.** Over 50% of anemic children live in low- and middle-income countries 12. **There is also a variation in anemia prevalence within low- and middle-income countries;** Repetition of sentence

**Under study design and setting**

1. No need to give district profile, better to shift under introduction.
2. Villages were randomly selected from a random sample of wards. I think the study was done in both urban and rural areas, so while using, use both village/wards.
3. How many villages and how many children from each villages/wards were selected is not clear. Also author has mentioned that the survey was done in villages, then wards also mentioned, is it a rural ward or urban, whether urban/rural children were selected is not clear. If so, how these children were selected from urban and rural areas were selected need to be mentioned.
4. Proportionate sampling should be done in order to give equal representation.

**Under Study population, sample size and sampling- **

1. It is mentioned that children whose mothers were not available on the day of data collection were excluded from the study as it was not possible to verify child information if next in kin or neighbor was interviewed. But in next para, it is mentioned that If the child’s mother was not at home, the research team visited the house for a minimum of three times before declaring that the participant could not be reached. This is not matching with above statement.
2. Ethical para should be written before analysis.
3. Why underweight prevalence is not given when weight has been measured.
4. In table 1, age of child mentioned is 6-26, pl correct 6-23.
5. Table 2, Time at complementary feeding, correct as age at CF.
6. While selecting reference category, positive variable should be consider as reference.
7. Adjusted analysis, which variables were adjusted in regression is not clear, which variable was removed first should be mentioned.

**Under discussion**

1. Prevalence in this study is similar to 38.8% among under-five children in Haiti, but higher than 26% in Cuba. The low prevalence in Cuba was associated to food-fortification interventions among other strategies.
2. Sentence need to rewrite properly  such as study by -----  observed 39% prevalence of anemia among under 5 year children in Haiti, while study by -------- observed lower (26%) prevalence of anemia in Cuba which may be due to food fortification being implemented among this study population. This practice could be one of the factors that leads to poor anemia status in children, sentence is not correct it is not poor anemia status but low hemoglobin levels.
3. Mothers of children aged 6–11 months in Australia did not receive quality anemia care, particularly nutrition advice about healthy foods and the minimum acceptable diet to the care givers- this should not be mentioned here as this study is not in Australia.
4. The high prevalence of anemia among infants in this study is of concern. Interventions such as iron supplementation, food fortification and dietary diversification and management of childhood illnesses in this setting should be targeted towards mothers and children less than two years. There were no significant differences in the prevalence of anemia by sex of the child in this study which is consistent to findings from other studies- there is no continuity of statement first sentence should deleted as it is there in last para.
5. Sentence writing needs to be changed, should take help from English speaking person who can write fluently.
6. From the study, no other factors except age of child was observed significant, other factors such as type of house, drinking water facility sanitary latrine, morbidities in previous  fortnight or one months if studied would have given more information, no new information /variable has been found or studied.

**Under conclusion**

1. Children in this age group were more likely to be anemic compared to those aged 24–59 months-this is of no use in this para first sentence itself is explanatory. In conclusion one has to mention that there is a need to explore other factors contributing to anemia as  IFS supplementation, morbidities during previous fortnight, sanitary latrine use and mothers hemoglobin status. In recommendation, it is mentioned IFA supplementation, but whether these children were supplemented IFA has not been studied although this is universal program. So recommendation should also be based on the finding of the study.
2. Spelling of complementary feeding should be checked.

**In general**

1. Discussion needs to be rewritten properly with continuity of sentence.

Is the work clearly and accurately presented and does it cite the current literature?

Yes

If applicable, is the statistical analysis and its interpretation appropriate?

Yes

Are all the source data underlying the results available to ensure full reproducibility?

Yes

Is the study design appropriate and is the work technically sound?

Partly

Are the conclusions drawn adequately supported by the results?

Yes

Are sufficient details of methods and analysis provided to allow replication by others?

Yes

Reviewer Expertise:

Public Health Nutrition

I confirm that I have read this submission and believe that I have an appropriate level of expertise to confirm that it is of an acceptable scientific standard, however I have significant reservations, as outlined above.

# Response

**Published:** 2021-10-20

**Authors:** Innocent Mboya

**Affiliations:** Lund University, Sweden

Dear Meshram, 

 Thank you for taking time to review our manuscripts. The manuscript has been revised based on your suggestions as explained below. 

**Comments:**

- *Introduction: Globally, about 43% of children under-five are anemic, and there is a marked variation in the prevalence of anemia between lowand middle-income countries. Over 50% of anemic children live in low- and middle-income countries 12. There is also a variation in anemia prevalence within low- and middle-income countries; Repetition of sentence*
- **Response: **The paragraph has been revised to avoid repetitions.
- *Under study design and setting: No need to give district profile, better to shift under introduction.*
- **Response:** The district profile is briefly discussed here to give a reader the context with respect to setting where the study was conducted. The background section focused on describing the problem given the current literature and showing the gap that necessitated the conduct of this study. For this reason, the district profile fits best in the methods section than in the introduction. The text has been slightly revised to avoid repetitions.
- *Villages were randomly selected from a random sample of wards. I think the study was done in both urban and rural areas, so while using, use both village/wards.*
- **Response: **Rombo is considered a rural district of Kilimanjaro region. However, participants were asked whether they considered the place of their residence as urban or rural. Those who resided in urban areas were mostly from the district’s centre (mostly referred by locals as urban or town). The key services such as the district’s commissioner’s office/headquarter, district hospital, among others are in this setting. In addition, wards are largest administrative units and contains several villages.
- *How many villages and how many children from each villages/wards were selected is not clear. Also author has mentioned that the survey was done in villages, then wards also mentioned, is it a rural ward or urban, whether urban/rural children were selected is not clear. If so, how these children were selected from urban and rural areas were selected need to be mentioned.*
- **Response:** Two villages were randomly selected from a random sample of wards. Also, a systematic random sampling was used to select households. Changes have been made to in the study population, sample size and sampling to make this more specific and clearer. Regarding urban vs rural, please see the response above.
- *Proportionate sampling should be done in order to give equal representation.*
- **Response: **More than half of all wards in the district were included in this study. For this reason, data from this study are representative of the district population. However, the findings from this study may not be generalized to other districts in Kilimanjaro region and the entire country. This has been added in the last paragraph of the discussion section detailing the study strengths and limitations.
- *Under Study population, sample size and sampling - It is mentioned that children whose mothers were not available on the day of data collection were excluded from the study as it was not possible to verify child information if next in kin or neighbor was interviewed. But in next para, it is mentioned that If the child’s mother was not at home, the research team visited the house for a minimum of three times before declaring that the participant could not be reached. This is not matching with above statement.*
- **Response:** The reviewer comment is acknowledged. The paragraph has been revised to merge and link this information.
- *Ethical para should be written before analysis.*
- **Response: **The ethics paragraph has been repositioned. 
- *Why underweight prevalence is not given when weight has been measured.*
- **Response: **Underweight results has been included in the descriptive statistics.
- *In table 1, age of child mentioned is 6-26, pl correct 6-23.*
- **Response:** Child age categories has been corrected in Table 1.
- *Table 2, Time at complementary feeding, correct as age at CF.*
- **Response: **Time at complementary feeding changed to Age at complementary feeding in Age 2 and 3.
- *While selecting reference category, positive variable should be consider as reference.*
- **Response: **We thank the reviewer for this comment. The initial analysis considered the positive category as the reference.
- *Adjusted analysis, which variables were adjusted in regression is not clear, which variable was removed first should be mentioned.*
- **Response: **Stepwise regression method was used to select variables to include in the adjusted analysis at 10% threshold level. Age of the child remained the only significant predictor of anemia at this stage. Maternal age, alcohol use (statistically significant in the crude analysis), sex of the child, and child’s nutritional characteristics, specifically exclusive breastfeeding, wasting, and stunting were considered potential confounders, hence included in the final model. This information has been added in the data analysis section.
- *Under discussion: Prevalence in this study is similar to 38.8% among under-five children in Haiti, but higher than 26% in Cuba. The low prevalence in Cuba was associated to food-fortification interventions among other strategies. Sentence need to rewrite properly  such as study by -----  observed 39% prevalence of anemia among under 5 year children in Haiti, while study by -------- observed lower (26%) prevalence of anemia in Cuba which may be due to food fortification being implemented among this study population.*
- **Response: **The sentences have been revised accordingly.
- *This practice could be one of the factors that leads to poor anemia status in children, sentence is not correct it is not poor anemia status but low hemoglobin levels.*
- **Response: **The sentence has been corrected.
- *Mothers of children aged 6–11 months in Australia did not receive quality anemia care, particularly nutrition advice about healthy foods and the minimum acceptable diet to the care givers- this should not be mentioned here as this study is not in Australia.*
- **Response: **The sentence has been revised and included the recommended best practices from this study.
- *The high prevalence of anemia among infants in this study is of concern. Interventions such as iron supplementation, food fortification and dietary diversification and management of childhood illnesses in this setting should be targeted towards mothers and children less than two years. There were no significant differences in the prevalence of anemia by sex of the child in this study which is consistent to findings from other studies- there is no continuity of statement first sentence should deleted as it is there in last para.*
- **Response: **The first two sentences have been deleted to ensure continuity of sentences in this paragraph.
- *Sentence writing needs to be changed, should take help from English speaking person who can write fluently.*
- **Response: **The manuscript has been revised to correct for grammatical errors and structure of sentences.
- *From the study, no other factors except age of child was observed significant, other factors such as type of house, drinking water facility sanitary latrine, morbidities in previous  fortnight or one months if studied would have given more information, no new information /variable has been found or studied.*
- **Response: **The reviewer comment is acknowledged. Determinants of anemia are multi-factorial and are likely to differ across settings. The discussion section has also highlighted numerous other factors that were analysed but had no significant statistical association with anemia in children. Qualitative and quantitative studies, especially the methods employing longitudinal measurements may be relevant in this context to determine the factors associated with anemia in children to inform interventions. This recommendation has been added in the conclusion paragraph.
- *Under conclusion: Children in this age group were more likely to be anemic compared to those aged 24–59 months-this is of no use in this para first sentence itself is explanatory. In conclusion one has to mention that there is a need to explore other factors contributing to anemia as  IFS supplementation, morbidities during previous fortnight, sanitary latrine use and mothers hemoglobin status. In recommendation, it is mentioned IFA supplementation, but whether these children were supplemented IFA has not been studied although this is universal program. So recommendation should also be based on the finding of the study.*
- **Response: **The recommendation has been added as suggested.
- *Spelling of complementary feeding should be checked.*
- **Response: **The spelling errors have been checked and corrected.
- *In general: Discussion needs to be rewritten properly with continuity of sentence.*
- **Response: **The discussion section has been revised based also on the reviewer suggestions.
